# Evaluation of the incidence, characteristics, and outcomes of pediatric chronic critical illness

**DOI:** 10.1371/journal.pone.0248883

**Published:** 2021-05-28

**Authors:** Hilmi Demirkiran, Mehmet Kilic, Yakup Tomak, Tahir Dalkiran, Sadik Yurttutan, Murat Basaranoglu, Oguz Tuncer, Turan Derme, Arzu Esen Tekeli, Ilhan Bahar, Siddik Keskin, Hafize Oksuz

**Affiliations:** 1 Department of Anesthesiology and Reanimation, Faculty of Medicine, Van Yuzuncu Yil University, Van, Turkey; 2 Department of Pediatrics, Hisar Intercontinental Hospital, Istanbul, Turkey; 3 Anesthesiology and Intensive Care Unit, Faculty of Medicine, Sakarya University, Sakarya, Turkey; 4 Pediatric Intensive Care Unit, Necip Fazil City Hospital, Kahramanmaras, Turkey; 5 Department of Pediatrics, Division of Neonatology, Faculty of Medicine, Kahramanmaras Sutcu Imam University, Kahramanmaras, Turkey; 6 Department of Pediatrics, Division of Neonatology, Faculty of Medicine, Van Yuzuncu Yil University, Van, Turkey; 7 Department of Pediatrics, Division of Neonatology, Ministry of Health Ankara City Hospital, University of Health Sciences, Ankara, Turkey; 8 Internal Medicine Critical Care Unit, Ataturk Training and Research Hospital, Izmir Katip Celebi University, Izmir, Turkey; 9 Department of Biostatistics, Faculty of Medicine, Van Yuzuncu Yil University, Van, Turkey; 10 Department of Anesthesiology and Reanimation, Faculty of Medicine, Kahramanmaras Sutcu Imam University, Kahramanmaras, Turkey; Kaohsuing Medical University Hospital, TAIWAN

## Abstract

Our aim was to determine characteristics of children with chronic critical illness (CCI) admitted to the pediatric intensive care unit (PICU) of a tertiary care children’s hospital in Turkey. The current study was a multicenter retrospective cohort study that was done from 2014 to 2017. It involved three university hospitals PICUs in which multiple criteria were set to identify pediatric CCIs. Pediatric patients staying in the ICU for at least 14 days and having at least one additional criterion, including prolonged mechanical ventilation, tracheostomy, sepsis, severe wound (burn) or trauma, encephalopathy, traumatic brain injury, status epilepticus, being postoperative, and neuromuscular disease, was accepted as CCI. In order to identify the newborn as a chronic critical patient, a stay in the intensive care unit for at least 30 days in addition to prematurity was required. Eight hundred eighty seven (11.14%) of the patients who were admitted to the PICU met the definition of CCI and 775 of them (87.3%) were discharged to their home. Of CCI patients, 289 (32.6%) were premature and 678 (76.4%) had prolonged mechanical ventilation. The total cost values for 2017 were statistically higher than the other years. As the length of ICU stay increased, the costs also increased. Interestingly, high incidence rates were observed for PCCI in our hospitals and these patients occupied 38.01% of the intensive care bed capacity. In conclusion, we observed that prematurity and prolonged mechanical ventilation increase the length of ICU stay, which also increased the costs. More work is needed to better understand PCCI.

## Introduction

Many critically ill patients survive their first acute attacks, but they continue to live with a syndrome that requires long-term intensive care, known as chronic critical illness (CCI) [1]. Adult CCI is defined as permanent multi-system dysfunction syndrome. In this syndrome, intensive care interventions support patients during the acute phase of the life-threatening disease but cannot return them to a good state of health [1, 2]. These patients have significant hospital care burdens, high hospitalization costs, morbidity, and poor outcomes [3, 4]. Therefore, developing strategies to prevent CCI and reduce costs has become increasingly important [2].

In childhood, CCI occurs in children with prolonged length of stay (LOS), technology addiction, or multiple organ system involvement; the prevalence of CCI is increasing [5]. Technological advances in the last three decades have been extremely successful, offering children the best opportunity for survival and recovery after life-threatening traumas and diseases in the pediatric intensive care unit (PICU). However, this situation causes an increase in the number of children with chronic and life-threatening conditions in the patient population in the PICU [6]. These patients, also defined as pediatric chronic critical illness (PCCI), are children with complicated and LOS. Therefore, a multidisciplinary and collaborative team is needed for these patients. However, strategies to adapt acute care approaches to this growing population lag behind clinical demand [7]. The definition of PCCI, which includes both babies and children, has not been established, thereby preventing accurate analysis of children with CCI. CCI has social and financial burdens for patients, families, and the healthcare system.

Although PCCI has been discussed extensively worldwide, it has not been studied in Turkey. The aim of this study is to determine characteristics and clinical results of PCCI in the three separate PICU and neonatal ICU (NICU).

## Materials and methods

This multicenter retrospective study was conducted in the NICU and PICU of Van Yuzuncu Yıl, Kahramanmaras Sutcu Imam, and Sakarya Universities in Turkey between 1 November 2017 and 31 October 2018, with the Non-interventional Clinical Research Ethics Committee Approved number of 21.09.2017 / 10. Ethics committee waived patient informed consent on the study. Sakarya University has 33 NICU and 8 PICU beds out of a total bed number of 970 and pediatric clinic bed number of 72, respectively. Kahramanmaras Sutcu Imam University has 30 NICU and 10 PICU beds out of a total bed number of 600 and pediatric clinic bed number of 55. Van Yuzuncu Yıl University has 45 NICU and 15 PICU beds out of a total bed number of 662 and pediatric clinic bed number of 32, respectively.

### Patients

In this study, medical records of pediatric patients from birth to 17 years of age in the NICU and PICU between January 2014 and December 2017 were examined. The patients were divided into two groups as neonatal between 0–28 days and pediatric patients between 28 days and 17 years. The pediatric group was then divided into two subgroups, infant (1–11 months) and pediatric (1–17 years). Separate criteria were set to identify neonatal and pediatric CCIs. Pediatric patients staying at the ICU for at least 14 days and having at least one additional criterion were accepted as CCI (prolonged mechanical ventilation, where prolonged mechanical ventilation was defined as mechanical ventilation for ≥ 6 hours/day for ≥ 21 consecutive days) [8], tracheostomy, sepsis, severe wound (burn) or trauma, encephalopathy (post-resuscitation syndrome, asphyxia, intracranial bleeding, metabolic disease, traumatic brain injury, status epilepticus, postoperative (cardiac and non-cardiac), and neuromuscular disease. In order to identify the newborn as a chronic critical patient, a stay in the intensive care unit for at least 30 days in addition to the prematurity was required. The data obtained from all hospitals were checked by one physician and defined as PCCI. This study registration number at clinicaltrials.gov is **NCT0330851.**

The demographic characteristics of the patients, primary admission diagnoses, and clinical outcomes were evaluated according to the definition of CCI using standard statistics. To calculate the cost in US dollars, the average annual exchange rate between the US dollar and Turkish Lira (TL) was applied based on data obtained from the Central Bank of the Republic of Turkey (i.e., US$1: 2.15, 2.10, 2.30, and 3.60 TL for 2014, 2015, 2016, and 2017, respectively).

### Statistical analysis

The data were evaluated using the IBM SPSS Statistics Standard Concurrent User V 25 (IBM Corp., Armonk, New York, USA) statistical package program. Descriptive statistics are given as number of units (n), percentage (%), mean, standard deviation, median, first and third quartiles, and minimum and maximum values. Risk factors that are thought to have an effect on mortality were evaluated with the univariate and multivariate Cox proportional hazard model. Backward Wald elimination method was used to create the final model in multivariate analysis. Hazard rate values are given with 95% confidence intervals. For Cox regression analysis, p <0.10 was considered statistically significant.

## Results

Out of 7961 PICU admissions during the study period, a total of 887 (11.14%) met the consensus definition for CCI. The reasons for hospitalization of these patients are shown in S1 Table.

Table 1 shows the demographic and clinical features of the patients. As seen in Table 1, 477 (53.8%) of the patients included in the study were hospitalized at Van Yuzuncu Yıl University. There was no difference between the gender of the patients, and 672 (75.8%) of the patients were the neonatal group. 32.6% of the patients with CCI were premature and 678 (76.4%) patients were exposed to prolonged mechanical ventilation. While 464 (52.3%) of patients did not have any comorbidities, 409 (46.1%) had one comorbidity. 775 (87.3%) of the treated patients were discharged to their homes. CCI patients occupied 38.01% of the intensive care bed capacity.

**Table 1:** Demographic characteristics of pediatric chronic critical patients for 2014–2017.

Variable	*N (*%)
**Center**	
Sakarya University	255 (28.7)
Kahramanmaras Sutçu İmam University	155 (17.5)
Van Yuzuncu Yıl University	477 (53.8)
**Treatment Year**	
2014	217 (24.5)
2015	202 (22.8)
2016	231 (26.0)
2017	237 (26.7)
**Gender**	
Female	430 (48.5)
Male	457 (51.5)
**Age**	
Neonatal (<1 month)	672 (75.8)
Infant (1–11 months)	181 (20.4)
Pediatric (1–17 years)	34 (3.8)
**Prematurity**	289 (32.6)
**Prolonged Mechanical Ventilation**	678 (76.4)
**Central Nervous System**	145 (16.3)
**Sepsis**	109 (12.3)
**Postoperative**	85 (9.6)
**Tracheostomy**	17 (1.9)
**Comorbidity Number**	
Zero	464 (52.3)
One	409 (46.1)
Two	14 (1.6)
**Cardiovascular**	82 (9.2)
**Respiratory**	37 (4.2)
**Kidney**	14 (1.6)
**Gastrointestinal**	37 (4.2)
**Hematology-Immunodeficiency**	100 (11.3)
**Metabolic**	39 (4.4)
**Growth Retardation**	31 (3.5)
**Last Condition Of The Patient**	
Dead	63 (7.1)
Complete recovery	775 (87.3)
Partial recovery	49 (5.5)

In Table 2, the costs were compared by years. Since the distribution of costs has a skewed distribution, logarithmic transformation was applied to the data and comparisons were made over the transformed data. Since the comparison is made over logarithmic values, the geometric mean and 95% confidence limits of the geometric mean are given as summary statistics. According to Table 2, total costs in TL differ between years. Total cost values for 2015 are statistically lower than other years, and total cost values for 2017 are statistically higher than other years. ICU costs in dollars did not differ statistically by years.

**Table 2:** Comparison of costs by years.

	YEARS				Test Statistics	
	2014	2015	2016	2017	*F*	*p*
	*GM*	*GM*	*GM*	*GM*		
	%95 *CI*	%95 *CI*	%95 *CI*	%95 *CI*		
Total Cost (TL)	31848.3	25997.4	32866.4	40727.3	**20.811**	**<0.001**
	(29079.2–34881.2)^*a*^	(23726.9–28485.3)^*b*^	(30811.8–35058.1)^*a*^	(37986.1–43666.3)^*c*^		
Total Cost (Dollar)	14813.2	12379.7	14289.7	11313.1	**10.090**	**<0.001**
	(13525.2–16223.7)^*a*^	(11298.5–13564.4)^*b*^	(13396.4–15242.6)^*a*^	(10551.7–12129.5)^*b*^		
ICU Cost (TL)	8622.3	7628.1	7282.7	11769.7	**6.349**	**<0.001**
	(7181.1–10352.7)^*a*^	(6485.6–8971.9)^*a*^	(6163.7–8605.1)^*a*^	(9872.6–14031.2)^*b*^		
ICU Cost (Dollar)	4010.4	3632.4	3166.4	3269.3	1.509	0.211
	(3340.1–4815.2)	(3088.4–4272.3)	(2679.8–3741.3)	(2742.4–3897.5)		
Hospital Duration	37.2	36.7	38.4	44.1	**6.897**	**<0.001**
	(34.7–39.9)^*a*^	(34.6–38.9)^*a*^	(36.4–40.4)^*a*^	(41.2–46.9)^*b*^		

TL: Turkish Lira; GM: Geometric mean; 95% CI: 95% confidence limits of the geometric mean; The a, b, and c superscript show if there is a difference between years. The years with the same letters are statistically similar.

Table 3 shows the risk factors affecting mortality by length of stay in the intensive care unit. Because of the low number of patients in the traumatic brain injury and oncological factors, these two factors were not included in multivariate models. Risk factors were evaluated both independently and corrected according to the total number of patients receiving (common variable-covariate) intensive care treatment for the year the patient was treated. When the values with and without correction according to Table 3 are analyzed, it is seen that risk factors are partially affected by the common variable but not significantly. When the factors were evaluated independently and without correction, the infant group was observed to have a 3.9 times higher mortality risk than the neonatal group. The presence of encephalopathy increased mortality 5.5 times and the risk of mortality decreased with increasing prolonged mechanical ventilation (HR = 0.974; p <0.001).

**Table 3:** Hazard rate and adjusted hazard rate (HR) estimations for risk factors on survival using univariate Cox proportional hazard model.

Variable	Mortality		Test Statistics		Adjusted Test Statistics*	
	No *n* (%)	Yes *n* (%)	*p*	*HR* (95% *CI* for *HR*)	*p*	*HR* (95% *CI* for *HR*)
**Gender**						
Female*	402 (93.5)	28 (6.5)		1		1
Male	422 (92.3)	35 (7.7)	0.492	1.190 (0.724–1.957)	0.495	1.189 (0.723–1.956)
**Age**						
Neonatal (<1 month)	632 (94.0)	40 (6.0)		1		1
Infant (1–11 months)	160 (88.4)	21 (11.6)	**<0.001**	3.862 (2.268–6.575)	**<0.001**	5.214 (2.775–9.797)
Pediatric (1–17 years)	32 (94.1)	2 (5.9)	0.260	2.267 (0.545–9.422)	0.129	3.117 (0.717–13.546)
**Prematurity**						
No	275 (95.2)	49 (8.2)		1		1
Yes	549 (91.8)	14 (4.8)	**0.019**	2.044 (1.127–3.707)	**0.018**	2.059 (1.134–3.740)
**Prolonged Mechanical Ventilation**						
No	202 (96.7)	7 (3.3)		1		1
Yes	622 (91.7)	56 (8.3)	0.663	0.837 (0.376–1.863)	0.672	0.840 (0.376–1.878)
**Traumatic Brain Injury**						
No	822 (93.0)	62 (7.0)		1		1
Yes	2 (66.7)	1 (33.3)	**0.017**	11.241 (1.543–81.903)	**0.018**	11.204 (1.518–82.699)
**Encephalopathy**						
No	767 (94.0)	49 (6.0)		1		1
Yes	57 (80.3)	14 (19.7)	**<0.001**	5.478 (3.019–9.940)	**<0.001**	5.481 (3.019–9.950)
**Heavy Wound**						
No	822 (92.9)	63 (7.1)	-	-	-	-
Yes	2 (100)	0 (0.0)				
**Sepsis**						
No	719 (92.4)	59 (7.6)		1		1
Yes	105 (96.3)	4 (3.7)	0.309	0.590 (0.214–1.630)	0.299	0.582 (0.210–1.615)
**Status Epilepticus**						
No	808 (92.8)	63 (7.2)	-	-	-	-
Yes	16 (100)	0 (0.0)				
**Postoperative**						
No	743 (92.6)	59 (7.4)		1		1
Yes	81 (95.3)	4 (4.7)	0.531	0.723 (0.262–1.994)	0.533	0.724 (0.263–1.997)
**Neuromuscular Disease**						
No	771 (92.7)	61 (7.3)		1		1
Yes	53 (96.4)	2 (3.6)	0.154	0. 358 (0.087–1.470)	0.154	0.358 (0.087–1.469)
**Tracheostomy**						
No	809 (93.0)	61 (7.0)		1		1
Yes	15 (88.2)	2 (11.8)	0.582	1.488 (0.362–6.118)	0.576	1.498 (0.363–6.172)
**Comorbidity**						
Zero	432 (93.1)	32 (6.9)		1		1
One	382 (93.4)	27 (6.6)	0.897	0.966 (0.577–1.617)	0.902	0.968 (0.578–1.622)
Two	10 (71.4)	4 (28.6)	**0.060**	2.789 (0.958–8.114)	**0.060**	2.787 (0.958–8.109)
**Cardiovascular**						
No	752 (93.4)	53 (6.6)		1		1
Yes	72 (87.8)	10 (12.2)	**0.072**	1.860 (0.946–3.657)	**0.070**	1.873 (0.951–3.692)
**Respiratory**						
No	787 (92.6)	63 (7.4)	-	-	-	-
Yes	37 (100)	0 (0.0)				
**Neuromuscular**						
No	745 (93.1)	55 (6.9)		1		1
Yes	79 (90.8)	8 (9.2)	0.714	1.150 (0.545–2.428)	0.716	1.149 (0.544–2.426)
**Renal**						
No	812 (93.0)	61 (7.0)		1		1
Yes	12 (85.7)	2 (14.3)	**0.098**	3.309 (0.803–13.642)	**0.097**	3.315 (0.804–13.667)
**Gastrointestinal**						
No	789 (92.8)	61 (7.2)		1		1
Yes	35 (94.6)	2 (5.4)	0.479	0.598 (0.144–2.482)	0.477	0.597 (0.144–2.478)
**Hematology-Immunodeficiency**						
No	728 (92.5)	59 (7.5)		1		1
Yes	96 (96.0)	4 (4.0)	0.533	0.724 (0.262–2.000)	0.540	0.726 (0.261–2.023)
**Metabolic**						
No	791 (93.3)	57 (6.7)		1		1
Yes	33 (84.6)	6 (15.4)	**0.009**	3.067 (1.317–7.141)	**0.010**	3.066 (1.314–7.152)
**Retinopathy**						
No	818 (92.8)	63 (7.2)	-	-	-	-
Yes	6 (100)	0 (0.0)				
**Growth Retardation**						
No	794 (92.8)	62 (7.2)		1		1
Yes	30 (96.8)	1 (3.2)	0.252	0.315 (0.044–2.274)	0.252	0.315 (0.044–2.276)
**Oncological**						
No	821 (93.1)	61 (6.9)		1		1
Yes	3 (60.0)	2 (40.0)	**0.004**	8.203 (1.997–33.700)	**0.004**	8.213 (1.980–34.071)
**Duration of Mechanical Ventilation**	-		**<0.001**	0.974 (0.965–0.982)	**<0.001**	0.974 (0.965–0.982)

*Adjusted for total number of patients receiving intensive care in the year the patient received treatment, *HR*: Hazard rate, *CI*: Confidence Interval

Factors affecting mortality, with and without correction, are given in Table 4. As a result of the analysis made according to the age group, encephalopathy, cardiovascular disease, and prolonged mechanical ventilation were determined as factors that had an effect on mortality.

**Table 4:** Hazard rate and adjusted hazard rate (HR) estimations for risk factors on survival using univariate Cox proportional hazard model.

	Test Statistics				Adjusted Test Statistics			
	*β*	*SE*	*p*	*HR* (95% *CI* for *HR*)	*β*	*SE*	*p*	*HR* (95% *CI* for *HR*)
**Age**								
Neonatal (<1 month)				1				1
Infant (1–11 months)	0.858	0.290	0.003	2.357 (1.336–4.160)	0.975	0.356	0.006	2.652 (1.320–5.327)
Pediatric (1–17 years)	-0.441	0.772	0.568	0.643 (0.142–2.923)	-0.308	0.807	0.702	0.735 (0.151–3.570)
**Encephalopathy**								
No				1				1
Yes	1.285	0.320	<0.001	3.614 (1.930–6.767)	1.262	0.322	<0.001	3.533 (1.879–6.641)
**Cardiovascular**								
No				1				1
Yes	0.669	0.352	0.058	1.953 (0.979–3.896)	0.647	0.355	0.068	1.910 (0.952–3.829)
**Duration of Mechanical Ventilation**	-0.021	0.005	<0.001	0.979 (0.970–0.988)	-0.021	0.005	<0.001	0.979 (0.970–0.988)

*Adjusted for total number of patients receiving intensive care in the year the patient received treatment, *HR*: Hazard rate, *CI*: Confidence Interval, *SE*: Standard error

In Table 5, the duration of ICU stay of patients with CCI is compared. Traumatic brain injury and severe wound categories were not included in the comparison due to the small number of patients. According to Table 5, the duration of ICU stay of those with prematurity and prolonged mechanical ventilation was significantly higher than those not prematurity and those with shorter mechanical ventilation. The length of stay in patients with encephalopathy and sepsis in the ICU was significantly lower than those without encephalopathy or sepsis. The differences in other CCIs were not statistically significant.

**Table 5:** Comparison of ICU stay by CCI presence.

		ICU Duration	Test Statistics	
CCI	*n*	*GM* (%95 *CI*)	*t*	*p*
**Prematurity**			4.882	<0.001
No	598	37.2 (35.7–38.7)		
Yes	289	43.7 (41.9–45.7)		
**Prolonged Mechanic Ventilation**			14.630	<0.001
No	209	26.9 (25.5–28.4)		
Yes	678	44.1 (42.6–45.4)		
**Encephalopathy**			4.692	<0.001
No	816	40.2 (38.9–41.4)		
Yes	71	29.6 (26.1–33.5)		
**Sepsis**			3.353	<0.001
No	778	40.2 (38.9–41.4)		
Yes	109	33.1 (29.6–36.9)		
**Status epilepticus**			1.432	0.153
No	871	39.4 (38.1–40.6)		
Yes	16	33.1 (23.6–46.6)		
**Postoperative**			1.316	0.189
No	802	39.5 (38.2–40.8)		
Yes	85	36.7 (33.0–41.0)		
**Neuromuscular Disease**			1.616	0.111
No	832	38.9 (37.7–40.2)		
Yes	55	44.2 (37.8–51.6)		
**Tracheostomy**			1.019	0.308
No	870	44.1 (32.7–59.3)		
Yes	17	39.1 (37.9–40.4)		

GM: Geometric mean; 95% CI: 95% confidence limits of the geometric mean.

## Discussion

In the intensive care units in developed countries, although most critically ill adults recover, 5–10% of them go from acute critical illness to permanent, and, in some cases, to CCI [9, 10]. Permanent critical disease is characterized as a disease associated with chronic low-level inflammation and organ failure that cannot be directly associated with the main cause of intensive care hospitalization [11, 12]. Patients with CCI stay in their ICUs for a long time and in most cases continue to need prolonged mechanical ventilation [1, 13]. Annual costs for this disease group in the United States is exceed $25 billion [2]. Clinical management plans and general care goals of patients with permanent or CCI require focusing on different topics such as relief of symptoms, rehabilitation, planning of discharge, and, in some cases, discontinuation of ventilation [14]. Therefore, in order to achieve these goals, strategies that focus on feasible care processes, where clinicians and decision makers have direct control, to improve patient and family experience and clinical outcomes need to be developed and implemented [12].

For this, a standard definition of CCI should be made. Various definitions for CCI include ICU stay time, prolonged mechanical ventilation, or tracheostomy. The use of a uniform definition for adult CCI is mandatory both to strengthen medical information about these patients and to guide health policy [11, 13]. According to an earlier definition, adult CCI patient must have been hospitalized for at least 8 days in an ICU and must have at least one of the following conditions: multiple organ failure, serious wounds, prolonged mechanical ventilation, tracheostomy, or sepsis/other serious infection [15]. Providing a consistent definition of CCI allows both clinicians and families to recognize the transition from acute intensive care disease to CCI. The number of people with chronic diseases that start in childhood is gradually increasing [16]. The benefits of a uniform definition with clear boundaries for CCI are not yet available for pediatric patients. Prolonged mechanical ventilation, tracheostomy, and ICU stay, which are the distinguishing hallmarks of adult CCI, are not definitive features for PCCI because respiratory insufficiency requiring prolonged mechanical ventilation is routine for many neonatal conditions. The expected duration of mechanical ventilation and NICU stay varies greatly depending on the degree of prematurity, but is usually one month for many premature babies [15].

In most of the studies, CCI was defined as only a chronic condition that would require a stay in ICU for more than 28 days [17–19]. Nine organ systems that cause this chronic condition have been identified including cardiovascular, respiratory, neuromuscular, congenital/genetic abnormalities, gastrointestinal, oncological, renal, metabolic/endocrinological, and hematological/immunological. CCI is not defined with a narrow frame and is left very broad. Despite the importance of PCCI, there is no clear information about the prevalence, consequences, and associated costs of this syndrome. To address this knowledge gap, we conducted a retrospective cohort study using PICU and NICU records from three university hospitals in Turkey. Our goals were to examine the prevalence, results, costs, and clinical features of PCCI patients in at least three university hospitals. Therefore, we created a definition for CCI from patients in the PICU and NICU. According to this definition, pediatric patients staying in the intensive care unit for at least 14 days and having at least one additional criterion, including prolonged mechanical ventilation, tracheostomy, sepsis, severe wound (burn) or trauma, encephalopathy, traumatic brain injury, status epilepticus, postoperative (cardiac and non-cardiac), and neuromuscular disease, were accepted as CCI. Since respiratory failure requiring mechanical ventilation is routine for many neonatal conditions, the expected mechanical ventilation and length of stay in the NICU has varied greatly depending on the degree of prematurity. However, it is usually greater than one month for most premature babies [15]. In order to identify the neonate as a chronic critical patient, the neonate was required to have stayed in the intensive care unit for at least 30 days in addition to being premature.

In our study, 11.14% of the patients who applied to the PICU met the definition for CCI. Ishihara et al [18] determined CCI at a rate of 58.15% in their study considering the broad-framed chronic condition. Similarly, Walter et al [20] found the CCI rate to be 51%. This ratio in our study indicates that a better understanding of the CCI definition is warranted. In this study, 289 of CCI patients were premature and 678 patients underwent prolonged mechanical ventilation. The lengths of ICU stay of patients with prolonged mechanical ventilation and prematurity were significantly higher than those without. 2017 was the year in which the length of ICU stay was the most by year. In addition, both intensive care cost and total cost were higher in 2017 compared to other years. We think that this increase in cost is due to the increase in ICU stay. In previous studies, they showed that the parameter most associated with total cost in the PICU was LOS [21, 22]. Despite the cost increase in 2017, the total dollar amount remained unchanged due to the increase in the Dollar-to-Turkish Lira exchange rate. These findings are important because our data show that premature children and children with prolonged mechanical ventilation do not only stay longer in the PICU but also consume far more hospital resources than full-term neonates. Prematurity and prolonged mechanical ventilation seem to be acceptable indicators of significant health complications as well as healthcare costs. In previous studies, cardiovascular and neuromuscular diseases have been reported as the most common cause of death in the critically ill pediatric patients [18, 23–25]. Namachivayam et al [19] observed that children who died in PICU were ventilated longer than survivors (34 days [25–50] versus 31 days [20–47]; p = 0.014). When we looked at the effect of CCI on mortality in our study, we found that having encephalopathy and cardiovascular disease increased mortality; however, prolonged mechanical ventilation decreased mortality. In addition to previous studies, we found that the mortality rate was higher in the infant group. While the association of encephalopathy and cardiovascular diseases with mortality is consistent with previous studies, the fact that prolonged mechanical ventilation reduces mortality contradicts the current literature. The rate of premature patients in our study group was high and mortality rates were lower than non-premature children. In addition, premature children needed mechanical ventilation for longer periods. For this reason, we think that the low mortality rate of premature patients affects the relationship between prolonged mechanical ventilation and mortality.

Understanding the characteristics of LOS patients in the PICU will make it easier for PICU healthcare providers to plan for critical care services [26]. A small number of children with critical illness require long-term admission to the PICU. LOS in PICU is generally considered to be longer than 12 to 30 days. Approximately 1 to 4.7% of total PICU admissions result in a long LOS [27–29]. The percentage of PICU admissions of children with CCI is 1.5 to 4.4% [19, 28, 30]. The proportion of children with CCI in the PICU is similar to the rate of LOS in PICU. This suggests that there may be a positive correlation between CCI and LOS. Mortality (15–40%) and long-lasting morbidity are higher in PICU patients with LOS compared to other patients [31]. In this study, it was observed that patients with CCI stayed in the hospital longer and mortality was high in some subgroups. Our findings are consistent with the results of previous studies.

In a meta-analysis, it was reported that the rate of adult CCI discharge to home varies between 20.2% and 21.8% [2]. In another study, this rate was similarly determined as 22% [32]. Namachian et al. [19] determined the discharge rate for Pediatric CCI to be 81.4%. In this study, the rate of discharge of patients with PCCI to home was 87.3%. As seen, the rate of healthy discharge of pediatric CCI is much higher than adult CCI. This is one of the important differences between Adult CCI and pediatric CCI. In addition, a study reported that patients with PCCI accounted for only 4.5% of the PICU population but 36% of the total bed days [33]. In this study, we similarly determined that pediatric CCI cases were 11.14% of the total PICU cases but accounted for 38.01% of the total bed use. For all these reasons, the management of PPCI is increasingly important.

Our study has some limitations. The sampling technique is not random but rather is based on manual chart review and referrals from other healthcare providers through interpersonal relationships. Since there is no common identification code for PCCI, data may not be complete, as some patients’ diagnoses of PCCI may have been missed. Another limitation is that this study does not include readmissions rates after discharge. One of the important limitations of the study is lack of a single nationwide database. Thus, this study does not contain data from public hospitals, private hospitals, and other university hospitals. Therefore, our findings will need to be confirmed by larger case studies.

In conclusion, early detection of NICU and PICU patients who may turn into CCI can help target interventions and resources for both patients and families. This is the first study conducted in Turkey on this issue. For this reason, we have determined some criteria for CCI in this study. According to the criteria, high incidence rates were observed for PCCI in our hospitals. CCI patients occupied 38.01% of the intensive care bed capacity. Moreover, we observed that prematurity and prolonged mechanical ventilation increase the length of ICU stay, which increases costs. More work is needed to define PCCI more clearly.

## Supporting information

S1 Data (XLSX)

S1 Table The reasons for hospitalization of CCI patients. (DOCX)
