# Supplementary material for: Evaluation of the incidence, characteristics, and outcomes of pediatric chronic critical illness
Source: PLoS One. 2021 May 28;16(5):e0248883. doi: 10.1371/journal.pone.0248883 (PMC8162636; doi:10.1371/journal.pone.0248883)
Supplement: S1 Table — (DOCX) [file pone.0248883.s002.docx]

**S1 Table:** The Reasons for Hospitalization of CCI Patients.

|  | ***N*** | **%** |
| --- | --- | --- |
| Prematurity | 155 | 17.5 |
| Respiratory Distress Syndrome | 117 | 13.2 |
| Respiratory Failure | 53 | 6.0 |
| Prematurity Anemia | 46 | 5.2 |
| Pneumonia | 30 | 3.4 |
| Neonatal Bacterial Sepsis | 27 | 3.0 |
| Other Cases With Low Birth Weight | 26 | 2.9 |
| Immune Deficiencies | 23 | 2.6 |
| Hydrocephalus | 22 | 2.5 |
| Premature Depolarization, Other And Unspecified | 21 | 2.4 |
| Patent Ductus Arteriosus | 19 | 2.1 |
| Bronchopulmonary Dysplasia That Begins In The Perinatal Period | 15 | 1.7 |
| Cerebral Palsy | 14 | 1.6 |
| Meningitis | 13 | 1.5 |
| Metabolic Disorders | 12 | 1.4 |
| Neonatal Jaundice, Unspecified | 11 | 1.2 |
| Nutritional Deficiencies | 10 | 1.1 |
| Nutritional Problems Of Neonatal | 10 | 1.1 |
| Ventricular Septal Defect | 9 | 1.0 |
| Bronchopneumonia, Unspecified | 8 | 0.9 |
| Other Cases Of Extremely Low Birth Weight | 8 | 0.9 |
| Acute Lymphoblastic Leukemia | 6 | 0.7 |
| Asphyxia | 6 | 0.7 |
| Atrial Septal Defect | 6 | 0.7 |
| Epilepsy | 6 | 0.7 |
| Cardiac Arrest | 6 | 0.7 |
| Congenital Hypotonia | 6 | 0.7 |
| Convulsions, Not Elsewhere Classified | 6 | 0.7 |
| Chronic Renal Failure | 6 | 0.7 |
| Prematurity + Respiratory Distress Syndrome | 6 | 0.7 |
| Undefined Chronic Respiratory Disease Starting From The Perinatal Period | 5 | 0.6 |
| Neonatal Anemia | 5 | 0.6 |
| Diabetes Mellitus | 4 | 0.5 |
| Hyperglycemia | 4 | 0.5 |
| Brain Edema | 3 | 0.3 |
| Febrile Convulsion | 3 | 0.3 |
| Developmental Retardation | 3 | 0.3 |
| Hypothyroidism, Unspecified | 3 | 0.3 |
| Intracerebral Hemorrhage | 3 | 0.3 |
| Heart Failure | 3 | 0.3 |
| Cardiac Murmur | 3 | 0.3 |
| Choanal Atresia | 3 | 0.3 |
| Septicemia | 3 | 0.3 |
| Spina Bifida | 3 | 0.3 |
| Neonatal Meconium Aspiration | 3 | 0.3 |
| Acute Renal Failure | 2 | 0.2 |
| Fever, Unspecified | 2 | 0.2 |
| Bronchopulmonary Dysplasia | 2 | 0.2 |
| Dyspnea | 2 | 0.2 |
| Birth Asphyxia, Unspecified | 2 | 0.2 |
| Encephalopathy | 2 | 0.2 |
| Hydronephrosis | 2 | 0.2 |
| Hypoplastic Left Heart Syndrome | 2 | 0.2 |
| Congenital Absence Of Other Defined Parts Of The Small Intestine, Atresia | 2 | 0.2 |
| Complications Of Intrauterine Interventions, Not Elsewhere Classified | 2 | 0.2 |
| Liver Failure | 2 | 0.2 |
| Malnutrition | 2 | 0.2 |
| Meningomyelocele | 2 | 0.2 |
| Fetus And Neonatal Affected By Oligohydramnios | 2 | 0.2 |
| Pneumothorax | 2 | 0.2 |
| Respiratory Syncytial Virus | 2 | 0.2 |
| Retinal Disorder | 2 | 0.2 |
| Rh Incompatibility Reaction | 2 | 0.2 |
| Volvulus | 2 | 0.2 |
| Neonatal Hypoglycemia | 2 | 0.2 |
| Abo Conflict Reaction | 1 | 0.1 |
| Adrenocortical Insufficiency | 1 | 0.1 |
| Adrenomedullary Hyperfunction | 1 | 0.1 |
| Acute Appendicitis | 1 | 0.1 |
| Acute Pancreatitis | 1 | 0.1 |
| Acute And Subacute Infective Endocarditis | 1 | 0.1 |
| Anus And Rectum Stenosis | 1 | 0.1 |
| Arnold-Chiari Syndrome | 1 | 0.1 |
| Atresia And Stenosis, Without Fistula | 1 | 0.1 |
| Falling From The Same Level | 1 | 0.1 |
| Benign And Harmless Heart Murmurs | 1 | 0.1 |
| Brain Malignant Neoplasm | 1 | 0.1 |
| Falling From One Level To Another | 1 | 0.1 |
| Localized Swelling, Mass And Lump Of The Neck | 1 | 0.1 |
| Bradycardia, Unspecified | 1 | 0.1 |
| Brucellosis | 1 | 0.1 |
| Nausea And Vomiting | 1 | 0.1 |
| Zinc Metabolism Disorders | 1 | 0.1 |
| Dextrocardia | 1 | 0.1 |
| Diabetes insipidus | 1 | 0.1 |
| Diabetic Mother Child Syndrome | 1 | 0.1 |
| Diabetic Retinopathy | 1 | 0.1 |
| Fetus And Neonatal Affected By Birth And Complications Of Birth | 1 | 0.1 |
| Congenital Malformation Syndromes Involving Limbs | 1 | 0.1 |
| Fetus And Neonatal Affected By Early Membrane Rupture | 1 | 0.1 |
| Fallot Tetralogy | 1 | 0.1 |
| Necrotizing Enterocolitis Of The Fetus And Neonatal | 1 | 0.1 |
| Gastroenteritis And Colitis, Non-Infective | 1 | 0.1 |
| Gastroschisis | 1 | 0.1 |
| Hemiplegia | 1 | 0.1 |
| Hemophagocytic Lymphohistiocytosis | 1 | 0.1 |
| Hyperlipidemia | 1 | 0.1 |
| Hypertension | 1 | 0.1 |
| Hypoglycemia | 1 | 0.1 |
| Hirschsprung's Disease | 1 | 0.1 |
| Immaturity | 1 | 0.1 |
| Side Effects Caused By Medicines And Therapeutic Agents | 1 | 0.1 |
| Extremely Low Birth Weight | 1 | 0.1 |
| Bleeding, Not Elsewhere Classified | 1 | 0.1 |
| Congenital Heart Block | 1 | 0.1 |
| Congenital Pneumonia | 1 | 0.1 |
| Congestive Heart Failure | 1 | 0.1 |
| Conjunctivitis, Unspecified | 1 | 0.1 |
| Conjunctivitis, Other | 1 | 0.1 |
| Craniopharyngeal Duct Malignant Neoplasm | 1 | 0.1 |
| Chronic Lung Disease | 1 | 0.1 |
| Laryngeal Web | 1 | 0.1 |
| Congenital Malformation Of The Larynx, Unspecified | 1 | 0.1 |
| Lumbar Vertebra Fracture | 1 | 0.1 |
| Malignant Neoplasm, Region Not Specified | 1 | 0.1 |
| Fracture Of The Mandible | 1 | 0.1 |
| Meningoencephalitis | 1 | 0.1 |
| Mitral Valve Insufficiency | 1 | 0.1 |
| Myasthenia Gravis And Other Myoneural Disorders | 1 | 0.1 |
| Mucocutaneous Lymph Node Syndrome | 1 | 0.1 |
| Mucopolysaccharidosis | 1 | 0.1 |
| Esophageal Atresia | 1 | 0.1 |
| Pleural Effusion | 1 | 0.1 |
| Fetus And Neonatal Affected By Polyhydramnios | 1 | 0.1 |
| Polycystic Kidney, Unspecified | 1 | 0.1 |
| Prematurity Retinopathy | 1 | 0.1 |
| Prematurity Retinopathy + Neonatal Jaundice | 1 | 0.1 |
| Prematurity Retinopathy + Nutritional Problems Of Neonatal | 1 | 0.1 |
| Prematurity + Pulmonary Hypertension | 1 | 0.1 |
| Primary Pulmonary Hypertension | 1 | 0.1 |
| Protein-Energy Malnutrition | 1 | 0.1 |
| Disease Of Pulmonary Vessels, Unspecified | 1 | 0.1 |
| Pulmonary Valve Stenosis | 1 | 0.1 |
| Purpura And Other Hemorrhagic Conditions | 1 | 0.1 |
| Renal Agenesis | 1 | 0.1 |
| Respiratory Distress Syndrome + Diabetic Mother Baby | 1 | 0.1 |
| Respiratory Distress Syndrome + Hydrocephalus | 1 | 0.1 |
| Respiratory Distress Syndrome + Pneumothorax | 1 | 0.1 |
| Respiratory Distress Syndrome + Neonatal Jaundice | 1 | 0.1 |
| Fever Of Unknown Origin And Other Origin | 1 | 0.1 |
| Cerebral Infarction | 1 | 0.1 |
| Digestive Organs Vague Or Unknown Behavioral Neoplasm | 1 | 0.1 |
| Subdural Hematoma | 1 | 0.1 |
| Subdural Hemorrhage (Non-Traumatic) | 1 | 0.1 |
| Drowning And Underwater | 1 | 0.1 |
| Sepsis Due To Unspecified Gram-Negative Organisms | 1 | 0.1 |
| Tachycardia, Unspecified | 1 | 0.1 |
| Injury To The Passenger In A Collision With Other And Unidentified Motor Vehicles In A Traffic Accident | 1 | 0.1 |
| Thrombocytopenia, Unspecified | 1 | 0.1 |
| Tubulointerstitial Disorders In Metabolic Diseases | 1 | 0.1 |
| Ulna Upper End Fracture | 1 | 0.1 |
| Urea Cycle Metabolism | 1 | 0.1 |
| Viral Encephalitis | 1 | 0.1 |
| Vitamin Deficiencies | 1 | 0.1 |
| Cleft Palate, Unspecified | 1 | 0.1 |
| Neonatal Encephalopathy | 1 | 0.1 |
| Neonatal Hypocalcemia | 1 | 0.1 |
| Neonatal Heart Failure | 1 | 0.1 |
